# Two alternative DNA extraction methods to improve the detection of *Mycobacterium-tuberculosis-*complex members in cattle and red deer tissue samples

**DOI:** 10.1186/s12866-016-0816-2

**Published:** 2016-09-15

**Authors:** Shari Fell, Stephanie Bröckl, Mathias Büttner, Anna Rettinger, Pia Zimmermann, Reinhard K. Straubinger

**Affiliations:** 1 Bacteriology and Mycology, Institute for Infectious Diseases and Zoonoses, Department of Veterinary Sciences, Faculty of Veterinary Medicine, LMU Munich, Veterinaerstr. 13, 80539 Munich, Germany; 2 Bavarian Health and Food Safety Authority, Veterinaerstr. 2, 85764 Oberschleißheim, Germany

**Keywords:** Bovine tuberculosis, MTC, DNA extraction, Magnetic capture, *Mycobacterium caprae*

## Abstract

**Background:**

Bovine tuberculosis (bTB), which is caused by *Mycobacterium bovis* and *M. caprae*, is a notifiable animal disease in Germany. Diagnostic procedure is based on a prescribed protocol that is published in the framework of German bTB legislation. In this protocol small sample volumes are used for DNA extraction followed by real-time PCR analyses. As mycobacteria tend to concentrate in granuloma and the infected tissue in early stages of infection does not necessarily show any visible lesions, it is likely that DNA extraction from only small tissue samples (20–40 mg) of a randomly chosen spot from the organ and following PCR testing may result in false negative results. In this study two DNA extraction methods were developed to process larger sample volumes to increase the detection sensitivity of mycobacterial DNA in animal tissue.

The first extraction method is based on magnetic capture, in which specific capture oligonucleotides were utilized. These nucleotides are linked to magnetic particles and capture *Mycobacterium-tuberculosis*-complex (MTC) DNA released from 10 to 15 g of tissue material. In a second approach remaining sediments from the magnetic capture protocol were further processed with a less complex extraction protocol that can be used in daily routine diagnostics. A total number of 100 tissue samples from 34 cattle (*n* = 74) and 18 red deer (*n* = 26) were analyzed with the developed protocols and results were compared to the prescribed protocol.

**Results:**

All three extraction methods yield reliable results by the real-time PCR analysis. The use of larger sample volume led to a sensitivity increase of DNA detection which was shown by the decrease of Ct-values. Furthermore five samples which were tested negative or questionable by the official extraction protocol were detected positive by real time PCR when the alternative extraction methods were used. By calculating the kappa index, the three extraction protocols resulted in a moderate (0.52; protocol 1 vs 3) to almost perfect agreement (1.00; red deer sample testing with all protocols).

**Conclusion:**

Both new methods yielded increased detection rates for MTC DNA detection in large sample volumes and consequently improve the official diagnostic protocol.

**Electronic supplementary material:**

The online version of this article (doi:10.1186/s12866-016-0816-2) contains supplementary material, which is available to authorized users.

## Background

*Mycobacterium bovis* and *M. caprae* are the causative agents of bovine tuberculosis (bTB) and belong among others to the *Mycobacterium-tuberculosis*-complex (MTC) [1, 2]. Its members cause tuberculosis (TB) in humans and other mammalian species worldwide and therefore hold zoonotic potential [3, 4].

Bovine tuberculosis can manifest as an acute or chronic infectious disease with a long subclinical phase and with the potential of reactivation after years [5]. In general, clinical signs depend on bacterial infectious dose and virulence, but also on the state of immune competence of the host and external influences [6]. The frequency of the presence and severity of clinical signs depends also on the host species, as in some species these are usually apparent, e.g. possums [7] and guinea pigs [8]. Most cattle that are infected do not develop clinical signs. In the early phase of infection, mycobacteria are phagocytosed by resident macrophages and may be eliminated. Alternatively, macrophages and/or dendritic cells (DC) will transport mycobacteria to the draining lymph node. With participation of interleukin-producing dendritic cells, macrophages, lymphocytes, neutrophils and epitheloid cells, the host immune system forms a tuberculoid granuloma [9]. This small granuloma prevents further spread of the pathogen to surrounding tissues and it can stay arrested for a long time. Bacilli within the center of the lesion do not multiply but may remain dormant and the resulting latent infection may persist for years [10]. Immunosuppression can disturb the balance between host control and agent dissemination, allowing replication of the silenced pathogen with reactivation and spread of the infection. They may knock out the host’s immune defense leading to bacteria spread to all tissues. Finally the mycobacteria will be shed via secretion/excretion and can find access to other susceptible hosts [5, 6]. However in animals, generalized manifestation is less common [11]. In contrast to the typical granuloma formation mostly in lymphoid tissue, non-visible lesions (NVL) may be present in many individuals after infection with bTB. These individuals show immune reactions in the tuberculin skin test in an early state of infection [9, 12]. Due to the localized manifestation of mycobacterial infection sometimes as only very small granuloma or tiny lesions, visual screening of carcasses after slaughter is challenging [13, 14].

Bovine tuberculosis is a notifiable animal disease in Germany. In 1996, Germany was declared “officially free of tuberculosis” (OTF) by the European Commission [15]. However, since 2008 until now, regular detection of *M. caprae* infections in cattle and red deer occurred in the Bavarian and Austrian alpine region [16, 17]. As a result, the national tuberculosis regulation was revised in 2009, 2012 and 2013 and an improvement of the control strategy was considered necessary.

The current diagnostic protocol in the framework of the government guided animal disease control [18] offers the tuberculin skin test (ante mortem), the gamma-interferon release assay (ante mortem), the bacteriological culture (post mortem), and a highly specific molecular detection method based on a real-time PCR protocol that is specific for MTC DNA detection in animal tissue (post mortem). In the official collection of recommended methods of the Friedrich-Loeffler-Institute (FLI) in Greifswald, Germany [19], the protocol for DNA extraction and the MTC specific real-time PCR are specified.

The guidelines require that DNA extraction is performed with 11 tissue samples per animal, which has been tested as bTB suspicious or positive using the single intradermal comparative cervical tuberculin test (SICCT) or showed suspicious lesions/granulomas after slaughter. One gram of each tissue sample is homogenized and 200 to 400 μl of the homogenate used for DNA extraction. However, due to the inhomogeneous distribution of *Mycobacteria* spp. in tissue material and the problem of NVL in the early stages of infection it is obvious that the use of small sample volumes for DNA extraction may result in false negative PCR results.

The aim of this study was the development of alternative DNA extraction methods, which allow the investigation of a larger sample mass (~2–20 g) to increase the probability of MTC DNA detection in animal tissue. First, a DNA extraction method based on magnetic capture was established. Magnetic capture of DNA is a method that has been used previously for the detection of other MTC members [20] and other in tissue inhomogenously scattered pathogens such as *Toxoplasma* spp. and eggs of *Echinococcus multilocularis* [21, 22]. In other recent studies, there are also other attempts to improve the diagnosis of infections caused by *M. bovis* on the basis of magnetic capture, not capturing specific DNA but applying an immunomagnetic method that concentrates mycobacteria with specific antibodies [23]. Secondly, we combined the lysis of a large sample volume with the less complex DNA extraction protocol from the official collection of recommended methods in order to simplify the application. As cultivation, usually the gold standard for bTB diagnostics, was not performed for each of the eleven tissue samples per animal, but only for cattle and red deer samples, which tested positive or equivocal by real-time PCR and/or showed visible lesions/granulomas, we decided to use the prescribed DNA extraction method (protocol 1) as reference method in our study. So thirdly, the results of the two newly established extraction methods were compared to the results obtained with the prescribed DNA extraction method. Comparison of all three protocols showed that processing of larger sample volumes increased the detection rate for *M. caprae* DNA. The new extraction protocols offer robust and easy to use tissue preparations to improve access to MTC DNA in larger sample masses and consequently reduce false negative results in routine bTB diagnostics.

## Methods

### Animal tissue

Organ samples originated from red deer and cattle that were sent to the local Food and Health Safety Authority in Oberschleißheim for bTB diagnosis as part of the regional bTB surveillance programs (cattle) or alpine monitoring (red deer) in 2013, 2014 and 2015 [16, 17]. The tissue samples from cattle were processed the same day of collection. Tissue samples from red deer were stored at −20 °C before diagnostics was initiated. DNA extraction was performed under BSL3 conditions and the heat inactivated extracts were further processed under BSL2 conditions. A total number of 100 tissue samples from 34 cattle (*n* = 74 tissue samples) and 18 red deer (*n* = 26 tissue samples; Tables 2 and 3), were examined for the presence of MTC DNA with the different DNA extraction methods and a subsequent real-time PCR.

Negative tissues samples used as controls for real-time PCR validation were collected from a bTB-negative cow slaughtered in the slaughterhouse in Munich in February 2013. This cow originated from a herd with no history of bTB infections. Organs did not show any pathological findings and DNA was extracted according to the prescribed protocol following the instructions of the official collection of recommended methods (protocol 1). Five intestinal lymph nodes, two lung lymph nodes and one retropharyngeal lymph node were chosen and confirmed negative for MTC by real-time PCR. Furthermore, pathological inconspicuous intestinal (*n* = 5) and lung (*n* = 5) lymph nodes of different weight (4.4 to 13.4 g) from five bTB-unsuspicious cattle from the slaughterhouse in Munich, collected in July 2014, were tested by DNA extraction protocol 1 and following real-time PCR and confirmed to be negative for MTC-specific DNA before they were used for the establishment of the new DNA extraction method based on magnetic capture.

### Mycobacteria culture and spiking of bTB uninfected bovine lymph nodes

*Mycobacterium bovis* BCG (ATCC No. 27289, DMSZ Braunschweig, Germany) was cultivated in BBL Middlebrook 7H9 broth with glycerol (Becton, Dickinson and Company, Franklin Lakes, USA) and Loewenstein-Jensen with glycerin and PACT (Merck, Darmstadt, Germany) for 6 to 8 weeks at 37 °C in the New Brunswick Galaxy 170 R incubator (Eppendorf AG, Hamburg, Germany) and confirmed as *M. bovis* BCG by the commercial Test Kit GenoType MTBC (Hain Lifescience GmbH, Nehren, Germany) following the manufacturer’s instructions. To establish the magnetic capture protocol, the MTC-negative lymph nodes (*n* = 10) were artificially spiked with five injections per lymph node each containing 100 μl of a *M. bovis* BCG suspension to mimic the inhomogeneous distribution of *Mycobacteria* spp. in animal tissue. The suspension was created using 1 ml phosphate buffered saline (PBS, according to Moore, 2001) and one loop of colony material from *M. bovis* BCG. Two samples of 1 g each from randomly chosen spots of ten spiked lymph nodes (20 samples in total) were processed according to the original DNA extraction protocol (protocol 1). Remaining tissue of the spiked lymph nodes was further processed separately according to the magnetic capture method (protocol 2).

### DNA extraction protocols

#### Protocol 1: standard mycobacterial DNA extraction from animal tissue according to the prescribed protocol of the official collection of recommended methods

With diagnostic samples of unknown microbial content, DNA extraction was executed with the DNeasy Blood and Tissue Kit (Qiagen, Hilden, Germany) according to the manufacture’s protocol “Purification of Total DNA from Animal Tissues (Spin-Column Protocol)” with the following modifications to suit the specific need for DNA extraction in the case of *Mycobacteria* spp. To increase the probability to detect MTC DNA in animal tissue, 1 g of each tissue sample was homogenized with 10 ml PBS. From the tissue suspension 200 to 400 μl were transferred to a 1.5-ml tube and centrifuged for 10 min at 10,000 × g (Eppendorf centrifuge 5430, Eppendorf AG, Hamburg, Germany). The pellet was re-suspended in 180 μl tissue lysis buffer (ATL buffer) and 20 μl proteinase K (20 mg/ml). Additionally, lysis time was increased to an overnight incubation. The next day, the tissue digest was heated for 20 min at 99 °C to inactivate proteinase K and potentially infectious material. After cooling to room temperature, 200 μl buffer AL were added. After centrifugation with buffer AW2 at 20,500 × g for 3 min, a DNeasy Mini spin column was placed into a 2-ml collection tube and centrifuged for an additional 1 min at 6800 × g to dry the DNeasy membrane completely. Eluted DNA was stored at −20 °C until real-time PCR was performed.

#### Protocol 2: magnetic capture assay

##### Tissue lysis and homogenization

Each diagnostic sample was weighed, cut into small pieces with a sterile surgical disposable scalpel (Braun, Aesculap AG, Tuttlingen, Germany), and put into a 50-ml screw cap tube (SuperClear, VWR International, Radnor, Pennsylvania), containing 20 of ¼” ceramic sphere beads (MP Biomedicals, Illkirch, France). The bovine tissue samples had an average weight of 5.3 g within a range of a minimum of 2 g to a maximum of 16 g. The tissue samples from red deer had an average weight of 5.1 g within a range of a minimum of 2 g to a maximum of 16 g. Depending on the weight on the sample, a defined volume of tissue lysis buffer (ATL buffer, Qiagen, Hilden, Germany) relative to the sample weight and proteinase K (20 mg/ml, Qiagen, Hilden, Germany) were added to the tube (volumes of buffers are shown in the Additional file 1: Table S1). Disruption and homogenization of the tissue samples to solubilize mycobacterial DNA were done using the FastPrep 96 homogenizer (MP Biomedicals, Solon, Ohio) equipped with the BIGFLEX 8 × 50 ml adapter. Samples were homogenized for 90 s with maximum speed at 1800 rpm. Then, tubes were placed in the Thermomixer C (Eppendorf AG, Hamburg, Germany) equipped with the Eppendorf SmartBlock 50-ml adapter and incubated overnight at 56 °C and 450 rpm. To inactivate proteinase K and potentially infectious bacteria, tubes were incubated twice at 99 °C and 450 rpm for 15 min with a short centrifugation step in between to spin down the material. After cooling down to room temperature, tissue digests were transferred into a new 50-ml screw cap tube (SuperClear, VWR International, Radnor, Pennsylvania). Tubes containing the pellet of used ceramic sphere beads were discarded. Samples were centrifuged (Eppendorf centrifuge 5810 R, Eppendorf AG, Hamburg, Germany) for 30 min at 5000 × g to spin down coarse materials. Supernatants (crude extract) were divided and placed into two 15-ml screw cap tubes (Sarstedt, Nümbrecht, Germany) for better handling. Subsequently, both 15-ml screw cap tubes were handled equally. Pellets were resuspended in 2 ml of distilled water and stored at −20 °C (*n* = 49).

##### Free biotin removal

To eliminate naturally occurring biotin from the sample high performance Streptavidin sepharose (GE Healthcare, Little Chalfont, United Kingdom) with a binding capacity of > 300 nmol/ml was used. Before use, a defined volume of the enzyme was washed three times (Additional file 1: Table S1). For that purpose, 1 ml of PBS was added to the enzyme and after mixing thoroughly the mixture spun down at 20,000 × g (Eppendorf centrifuge 5424) for 2 min. This process was repeated twice and thereafter the streptavidin sepharose was resuspended in the initial volume. The washed enzyme was added to the crude extract. Tubes were placed on a shaker (ST 5, CAT, M. Zipperer GmbH, Staufen, Germany) and incubated at room temperature for 30 min and 40 rpm to allow for streptavidin-biotin binding before centrifugation at 5000 × g for 10 min to remove the sepharose. Up to 10 ml of each supernatant were transferred into clean 15-ml tubes.

##### Hybridization of the biotinylated capture probe

For sequence-specific DNA capturing, 10 μl of each capture oligonucleotide (Cap1 to 4; 1 pmol/μl) were added to each supernatant. Sequences of these capture oligonucleotides are shown in Table 1. The solutions were heated at 95 °C for 15 min in the Thermomixer C (Eppendorf AG) equipped with the Eppendorf SmartBlock 15-ml adapter to denature the target DNA, followed by an incubation at 55 °C for 30 min and 300 rpm to allow hybridization between capture-oligonucleotide and MTC-DNA. Tubes were left to cool down to room temperature for 15 min on the shaker at 40 rpm.

**Table 1:** Oligonucleotide sequences of capture-oligonucleotides, real-time PCR primers and probes

No.		Name	Sequence 5′-3′	5′-label	3′-label
1	oligonucleotides for capturing MTC DNA: Cap 1 and Cap 2 are specific for Heli; Cap 3 and Cap 4 are specific for IS 1081	Cap 1	TTG ATC AGG TCG ACG ATG TAG	Biotin TEG	
		Cap 2	TCA CCA CCG ACA AAG CGT C	Biotin TEG	
		Cap 3	CTC TCG ACG TTC ATC GCC G	Biotin TEG	
		Cap 4	TGG CGG TAG CCG TTG CGC	Biotin TEG	
2	duplex real-time PCR specific for the hypothetical Helicase (Heli)	MTC-Heli 4 F	TTG ATC AGG TCG ACG ATG TAG		
		MTC Heli 4R	TCA CCA CCG ACA AAG CGT C		
		MTC Heli 4 FAM	TCA ACG ACC CCA ACG ACT GGT GC	FAM	BHQ1
3	duplex real-time PCR specific for the insertion sequence 1081 (IS 1081)	MTC IS 1081 5 F	CTC TCG ACG TTC ATC GCC G		
		MTC IS 1081 5R	TGG CGG TAG CCG TTG CGC		
		MTC IS 1081 5 FAM	ATT GGA CCG CTC ATC GCT GCG TTC	FAM	BHQ1
4	Duplex real-time PCR specific for ß-actin	ACT2 1030 F	AGC GCA AGT ACT CCG TGT G		
		ACT 1135 R	CGG ACT CAT CGT ACT CCT GCT T		
		ACT-1081 1105 Y	TCG CTG TCC ACC TTC CAG CAG ATGT	YakimaYellow	BHQ1

Primer and Probes, no. 2, 3 and 4 according to the Official Collection of Methods [19]

##### Capturing the target DNA

Per 15 ml tube 80 μl of M-270 Streptavidin Dynabeads (Invitrogen by Life technologies AS, Oslo, Norway, 10 mg/ml, 2.8 μm, binding capacity 650–1350 pmoles/mg beads) were washed three times in 1 ml binding and washing (B&W) buffer (5 mM Tris HCl pH 7.5, 0.5 mM EDTA pH 8.0, 1.0 M NaCl) according to the manufacturer’s instruction in a 2-ml tube. The beads were pelletized in a Dynal MPC-S magnet (Invitrogen by Life technologies AS) between the washing steps and resuspended in the initial volume of B&W buffer. The washed beads and defined volumes of 5 M NaCl (Additional file 1: Table S1) were added to each supernatant and the supernatants were incubated at room temperature for 60 min on the shaker at 40 rpm.

##### Washing of magnetic beads

The resultant complex of streptavidin bead and biotin labelled capture-oligonucleotide with potentially hybridized MTC DNA was isolated using the Dynal MPC-1 magnet (Invitrogen by Life technologies AS). For this purpose the two 15-ml tubes containing supernatants of the same tissue sample were placed in a MPC-1 magnet for 10 min. Then, supernatants were removed with a disposable Pasteur pipette (230 mm, Brand GmbH & Co KG, Wertheim, Germany). The tubes were removed from the magnet and remaining beads on the inner tube wall were washed down with 5 ml of B&W buffer and transferred into new 5-ml tubes (Eppendorf AG, Hamburg, Germany). Magnetic bead isolation (5 min) and the following wash step were repeated in 5-ml and 1.5-ml tubes using 5 ml and 1 ml B&W buffer. Remaining beads were finally resuspended in 100 μl of distilled water. The bead suspension was heated to 100 °C for 10 min to release MTC DNA into solution. The tubes were then placed in the MPC-S magnet and resulting supernatants were immediately transferred to one clean 1.5-ml tube. Beads were discarded and supernatants were stored at −20 °C for real-time PCR analysis.

#### Protocol 3: DNA extraction from the sediments

Sediment suspensions produced as byproducts of the magnetic capture protocol (see “Protocol 2: magnetic capture assay, Tissue lysis and homogenization”) of 49 tissue samples were treated according to protocol 3. For this purpose, these sediments were thawed. Aliquots of 200 μl were subjected to DNA extraction by adding 200 μl AL-Buffer. The remaining steps of DNA extraction were performed as outlined in protocol 1.

### Measurement of DNA quality and quantity

As all samples from cattle and red deer had been processed in daily routine diagnostics, it is not possible to provide further information regarding DNA quantity and quality generated with protocol 1. All samples generated with protocol 2 and protocol 3 were checked for DNA content and quality in duplicate with the Eppendorf Biophotometer D30 (Eppendorf AG, Hamburg, Germany) and average values were calculated.

### Duplex real-time PCR according to the official collection of recommended methods

Briefly, two different target sequences were used to prove the presence of MTC DNA in the sample namely a hypothetical helicase (Heli) and an insertion element (IS) 1081. As internal control system for a successful DNA extraction, amplification of genomic DNA of the beta-actin (ß-actin) gene (tissue) was integrated in both PCRs. An overview of primers and probes used for amplification are shown in Table 1. Internal controls for real-time PCR validation were prepared as follows.

As positive FAM control, DNA extracted according to protocol 1 from a pure culture of *M. bovis* BCG (positive control), and as positive HEX control, DNA from bTB-free lymph nodes (EC, tested negative by our reference method, protocol 1) were also implemented into the detection protocol. Water (Carl Roth, Karlsruhe, Germany) was used as negative control..

Each DNA sample was evaluated in triplicates. The average of triplicate Ct-values was used for data interpretation. For each of the three primer-probe combinations, a primer-probe mix according to the official collection of recommended methods for better handling was designed (Heli and IS 1081: 10 pmol primer/μl + 1.25 pmol probe/μl; ß-actin: 2.5 pmol primer/μl + 1.25 pmol probe/μl). Twenty-five microliters reaction mixture consisted of 3.5 μl PCR water (Carl Roth, Karlsruhe, Germany), 12.5 μl of 2X QuantiTect Multiplex Master Mix (QuantiTect Multiplex PCR Kit no ROX, Qiagen, Hilden, Germany), 2.0 μl of primer-probe mix ß-actin, 2.0 μl of primer-probe mix either Heli or IS 1081, and 5.0 μl of template undiluted DNA. PCR amplification was performed in 96-well plates (Sarstedt, Nümbrecht, Germany) using a Stratagene Mx3005P thermal cycler instrument (Agilent Technologies, Santa Clara, California) with following cycling conditions: initial incubation step at 95 °C for 15 min to activate the DNA polymerase, followed by 45 amplification cycles that consisted of a denaturation step at 95 °C for 1 min, an annealing step at 60 °C for 30 s, and an extension step at 72 °C for 30 s. Fluorescence signals were collected in the annealing phase and were detected via the FAM and the HEX channel.

### Interpretation of real-time PCR results

All tissue samples and the positive control were required to produce ß-actin signals with a threshold cycle (Ct) of more than 21 in the HEX channel. If no signals (no Ct-value) were detected in the HEX channel (tissue DNA) and also in the FAM channel (MTC DNA), the real-time PCR was not evaluable. Hence, DNA isolation and/or PCR were repeated. Since negative control and positive control extracts did not contain tissue DNA, no Ct-values were expected in the HEX channel.

The MTC-specific target sequences Heli and IS 1081 were considered positive below a Ct-value of 39.00, calculated as the mean of a triplicate. If only one of the two target genes produced a sufficient signal, the sample was considered equivocal. If no Ct-value was generated, samples were interpreted as negative.

### Mycobacterial cultivation of field samples and characterization

All red deer and cattle that were tested MTC-positive or equivocal by real-time PCR after DNA extraction protocol 1 were further processed by culture under BSL3 conditions at the Bavarian Health and Food Safety Authority, Oberschleißheim, as described by Rettinger et al. [17]. As mentioned earlier, not all 11 tissue samples per animal were cultivated, but only samples with visible lesions/granulomas and all the tissue samples tested positive and equivocal by real-time PCR were chosen for further investigation. Single colonies of the culture material were used for species identification using GenoType MTBC (Hain Lifescience GmbH, Nehren, Germany).

### Microscopic examination of the sediments

#### Acid fastness/Ziehl-Neelsen staining technique

To proof whether mycobacteria can be found in the homogenized and inactivated sediments, Ziehl-Neelsen staining was carried out on four sediments, which were thawed from −20 °C to room temperature. Sediments were chosen according to their Ct-value for MTC-DNA. One retropharyngeal lymph node from cattle and one retropharyngeal lymph node from red deer were selected as positive organs (animal no. 18, Table 2 and no. 17, Table 3). Samples from animal no. 7 and no. 22 (Table 2) were used as negative samples. Ten microliters of sediment were spread with a sterile pipette tip on a microscope slide (Henry Schein, Melville, New York, USA). The preparation was air-dried, fixed by heat, stained with carbol fuchsin solution (Merck, Darmstadt, Germany) and allowed to stand for 5 min. The stained smear was washed with distilled water and decolorized with hydrochloric acid (Merck, Darmstadt, Germany) in ethanol for up to 30 s. The preparation was washed again with distilled water and counterstained with malachite green (oxalate) solution (Merck, Darmstadt, Germany) for 1 min. Additional rinsing was done with distilled water to remove excess color and the slide was allowed to air-dry. Slides were screened at 1,000X magnification (oil emersion objective) in a microscope (Leica DM5000B, Leica CTR5000, Leica Microsystems, Wetzlar, Germany). Acid fast bacilli appeared in red, tissue material in blue. A red, straight or slightly curved rod occurring singly or in a cluster indicated the presence of acid fast tubercle bacilli.

**Table 2:** Ct-values of the three DNA extraction protocols of the MTC-specific target genes Heli and IS 1081 generated with the field tissue samples of cattle

Animal No.	Tissue	Weight in grams	Protocol 1		Protocol 2		Protocol 3		Protocol 1	Protocol 2	Protocol 3
			Heli	IS 1081	Heli	IS 1081	Heli	IS 1081	Overall result per animal		
1	Lung	5	no ct	no ct	no ct	no ct	–	–	negative	negative	–
	Diaphragm	5	no ct	no ct	no ct	no ct	–	–			
2	Lung	5	no ct	no ct	no ct	no ct	–	–	negative	negative	–
3	Intestinal ln	3	no ct	no ct	no ct	no ct	–	–	negative	negative	negative
	Kidney	3	no ct	no ct	no ct	no ct	no ct	no ct			
	Kidney ln	2	no ct	no ct	no ct	no ct	no ct	no ct			
4	Intestinal ln	5	no ct	no ct	no ct	no ct	–	–	negative	negative	negative
	Liver	4	no ct	no ct	no ct	no ct	no ct	no ct			
5	Ln cervicalis superf.	8	no ct	no ct	no ct	no ct	no ct	no ct	negative	negative	negative
	Mediastinal ln	10	no ct	no ct	no ct	no ct	no ct	no ct			
	Kidney	7	no ct	no ct	no ct	no ct	–	–			
	Kidney ln	4	no ct	no ct	no ct	no ct	–	–			
6	Liver	5	no ct	no ct	no ct	no ct	–	–	negative	negative	negative
	Lung	2	no ct	no ct	no ct	no ct	no ct	no ct			
	Kidney	4	no ct	no ct	no ct	no ct	–	–			
7	Lung^a^	3	no ct	no ct	no ct	no ct	no ct	no ct	negative	negative	–
8	Lung ln	6	27.20	26.96	31.60	32.50	–	–	positive	positive	–
9	Retropharyngeal ln	3	37.99	37.58	36.93	37.64	–	–	positive	positive	–
10	Intestinal ln	4	31.10	30.54	28.87	29.32	31.85	32.08	positive	positive	positive
11	Liver ln	4	43.92	37.32	35.59	35.00	37.54	39.29	positive	positive	positive
	Lung	2	34.26	31.18	37.77	39.37	37.56	38.68			
12	Intestinal ln	5	28.21	26.69	31.10	31.50	–	–	positive	positive	–
13	Intestinal ln	6	29.23	28.02	38.42	39.25	35.53	35.65	positive	equivocal	positive
14	Intestinal ln	4	no ct	no ct	33.90	33.92	33.97	34.20	negative	positive	positive
15	Diaphragm	10	no ct	no ct	no ct	no ct	no ct	no ct	negative	negative	negative
	Diaphragm	10	no ct	no ct	no ct	no ct	no ct	no ct			
	Diaphragm	3	no ct	no ct	no ct	no ct	–	–			
16	Intestinal ln	3	35.31	35.60	no ct	no ct	33.41	34.74	positive	positive	positive
	Lung	3	no ct	no ct	no ct	no ct	no ct	no ct			
	Lung ln	3	29.71	30.38	30.45	31.05	30.56	30.90			
	Spleen	6	no ct	no ct	no ct	no ct	41.09	37.00			
	Kidney	8	no ct	no ct	no ct	no ct	no ct	no ct			
17	Intestinal ln	3	34.31	34.36	32.00	32.20	–	–	positive	positive	–
18	Liver	4	no ct	no ct	no ct	no ct	–	–	positive	positive	positive
	Lung^a^	5	26.53	27.10	24.80	24.86	26.28	26.73			
	Mediastinal ln	8	30.22	30.40	32.66	33.19	32.57	32.82			
	Spleen	5	no ct	no ct	34.50	35.70	–	–			
	Kidney	3	no ct	no ct	38.80	38.20	–	–			
	Retropharyngeal ln	2	no ct	no ct	ne	ne	–	–			
19	Mediastinal ln	3	no ct	no ct	no ct	no ct	no ct	no ct	negative	negative	negative
20	Intestinal ln	5	32.95	32.87	33.80	34.40	–	–	positive	positive	–
	Mediastinal ln	5	35.88	36.29	38.70	40.90	–	–			
	Retropharyngeal ln	4	no ct	no ct	no ct	no ct	–	–			
21	Lung ln	4	no ct	no ct	29.29	30.08	31.59	31.43	negative	positive	positive
22	Intestinal ln	8	no ct	no ct	no ct	no ct	no ct	no ct	negative	negative	negative
	Liver	6	no ct	no ct	no ct	no ct	no ct	no ct			
	Lung	6	no ct	no ct	no ct	no ct	–	–			
	Lung ln	9	no ct	no ct	no ct	no ct	–	–			
	Spleen	6	no ct	no ct	no ct	no ct	–	–			
	Kidney	9	no ct	no ct	no ct	no ct	–	–			
	Retropharyngeal ln^a^	5	no ct	no ct	no ct	no ct	no ct	no ct			
23	Kidney	2	no ct	no ct	ne	ne	ne	ne	negative	positive	equivocal
	Muscle	9	no ct	no ct	37.60	37.72	38.65	40.23			
	Palatine tonsil	3	no ct	no ct	39.57	41.83	39.17	39.14			
24	Udder ln	3	no ct	no ct	no ct	no ct	no ct	no ct	negative	negative	negative
25	Intestinal ln	2	no ct	no ct	32.92	34.22	31.61	31.05	negative	positive	positive
26	Lung ln	5	no ct	no ct	no ct	no ct	–	–	negative	negative	negative
	Rumen	5	no ct	no ct	no ct	no ct	no ct	no ct			
27	Lung	6	no ct	no ct	no ct	no ct	–	–	negative	negative	negative
	Thymus	16	no ct	no ct	no ct	no ct	no ct	no ct			
28	Lung	6	no ct	no ct	39.00	no ct	–	–	negative	negative	–
29	Pelvic ln	5	no ct	no ct	no ct	no ct	–	–	negative	positive	positive
	Mesentery	8	no ct	no ct	36.32	36.63	37.46	38.95			
	Kidney	6	no ct	no ct	no ct	no ct	–	–			
	Perintoneum	11	no ct	no ct	no ct	no ct	no ct	no ct			
30	Intestinal ln	3	no ct	no ct	34.14	34.49	38.22	38.39	negative	positive	positive
	Liver ln	5	no ct	no ct	no ct	no ct	–	–			
	Lung ln	6	no ct	no ct	no ct	no ct	–	–			
	Retropharyngeal ln	7	no ct	no ct	no ct	no ct	–	–			
31	Kidney	5	no ct	no ct	no ct	no ct			negative	negative	–
32	Lung	5	no ct	no ct	no ct	no ct	no ct	no ct	negative	negative	negative
	Lung ln	5	no ct	no ct	no ct	no ct	–	–			
33	Lung ln	6	no ct	no ct	no ct	no ct	–	–	negative	negative	–
34	Spleen	3	no ct	no ct	no ct	no ct	no ct	no ct	negative	negative	negative

*ln* lymph node, *ne* not evaluable

– not done

^a^microscopic examination of Ziehl-Neelsen-stained sediments

**Table 3:** Ct-values of the three DNA extraction protocols of the MTC-specific target genes Heli and IS 1081 generated with the field tissue samples of red deer

Animal No.	Tissue	Weight in grams	Protocol 1		Protocol 2		Protocol 3		Protocol 1	Protocol 2	Protocol 3
			Heli	IS 1081	Heli	IS 1081	Heli	IS 1081	Overall result per animal		
1	Intestinal ln	5	no ct	no ct	no ct	no ct	–	–	negative	negative	–
2	Intestinal ln	3	no ct	no ct	no ct	no ct	no ct	no ct	negative	negative	negative
3	Mesenteric ln	2	no ct	no ct	no ct	no ct	–	–	negative	negative	–
4	Intestinal ln	8	no ct	no ct	no ct	no ct	–	–	negative	negative	–
5	Intestinal ln	3	no ct	no ct	no Ct	no Ct	–	–	negative	negative	–
6	Peritoneum	2	no ct	no ct	no Ct	no Ct	–	–	negative	negative	–
7	Intestinal ln	4	no ct	no ct	no Ct	no Ct	41.90	no ct	negative	negative	negative
8	Mesenteric ln	3	35.63	34.79	ne	ne	–	–	positive	–	–
9	Intestinal ln	3	no ct	37.57	ne	ne	–	–	positive	positive	–
	Lung	5	26.27	26.14	27.00	27.20	–	–			
10	Intestinal ln	2	no ct	no ct	no Ct	no Ct	–	–	negative	negative	–
	Lung	3	no ct	no ct	ne	ne	–	–			
11	Intestinal ln	19	33.78	35.20	35.02	35.53	33.11	33.60	positive	positive	positive
	Retropharyngeal ln	3	no ct	39.54	38.09	37.96	41.77	41.11			
12	Intestinal ln	5	37.54	36.79	35.67	36.38	37.82	38.08	positive	positive	positive
	Retropharyngeal ln	11	no ct	no ct	38.82	39.81	37.03	36.81			
13	Lung	7	33.74	34.62	35.90	36.20	–	–	positive	positive	–
	Pleura	2	37.14	37.07	36.10	37.20					
	Palatine tonsil	3	35.49	36.08	33.20	33.10					
	Mass	8	34.03	38.53	32.80	33.10					
	Diaphragm	11	31.85	31.84	38.10	37.60					
14	Lung	3	no ct	no ct	no Ct	no Ct	no ct	no ct	negative	negative	negative
15	Retropharyngeal ln	8	no ct	no ct	no Ct	no Ct	no ct	39.92	negative	negative	negative
16	Intestinal ln	2	no ct	no ct	ne	ne	no ct	no ct	negative	–	negative
17	Retropharyngeal ln^a^	5	17.95	25.95	26.88	27.61	25.91	26.25	positive	positive	positive
18	Intestinal ln	2	no ct	no ct	no Ct	no Ct	no ct	no ct	negative	negative	negative

*ln* lymph node, *ne* not evaluable

– not done

^a^microscopic examination of Ziehl-Neelsen-stained sediments

#### Statistical analysis

Statistical analysis was conducted using MedCalc statistical software ver. 16.4.3 (MedCalc Software bvba, Ostend, Belgium). The agreement level between the overall results per animal for the three extraction protocols was assessed using the kappa (k) index and interpreted as follows: < 0.00 poor, 0.0–0.20 slight, 0.21–0.40 fair, 0.41–0.60 moderate, 0.61–0.80 substantial, 0.81–1.00 almost perfect agreement [24]. Protocol 1 was used as reference method and the results of protocol 2 and 3 were compared each to protocol 1 and to each other. Unevaluable results were not included in the calculation.

## Results

### *M. bovis* BCG cultivation and real-time PCR validation

*M. bovis* BCG, which was used as positive control for real-time PCR validation, grew to sufficient quantity after 5 weeks of incubation. Culture material was used for DNA extraction (protocol 1) and implemented as positive control showing average Ct-values of Ct_Heli_ = 18.30 (ß-actin + Heli) and Ct_IS 1081_ = 17.53 (ß-actin + IS 1081). There were no signals for ß-actin.

The MTC-negative lymph nodes used as extraction and HEX positive/FAM negative control were extracted in accordance to protocol 1. None of the used lymph nodes showed amplification results for Heli or IS 1081, but ß-actin averaged at Ct_ß-Actin_ = 19.42 (ß-actin + Heli) and Ct_ß-Actin_ = 18.84 (ß-actin + IS 1081).

### Establishing the magnetic capture protocol by utilizing artificially spiked lymph nodes

Twenty samples were processed according to protocol 1 and tested negative for MTC DNA (average Ct_ß-actin_ = 22.65) and only eight samples were MTC-positive (average Ct_ß-actin_ = 22. 89, Ct_Heli_ = 34.32, and Ct_IS 1081_ = 35.02). When protocol 2 was applied, all spiked lymph nodes (*n* = 10) turned out to be MTC-positive (100 %) and produced reliable amplification signals for MTC DNA (average Ct_Heli_ = 31.90, average Ct_IS 1081_ = 30.03). However, compared to protocol 1 less ß-actin was detected (average Ct_ß-actin_ = 28.65). Considering the fact that protocol 2 aimed specifically at MTC DNA and neglected host-specific genes, which were removed with the supernatants, no better Ct-values for ß-actin were expected.

### Evaluation of field samples processed according to protocols 1 to 3

All 100 tissue samples processed according to protocol 1 produced evaluable real-time PCR results and each tissue sample could consequently be classified as positive for MTC-specific DNA, negative or equivocal. Also the remaining tissue materials from cattle and red deer (*n* = 100), which were further processed according to protocol 2 (*n* = 100) and protocol 3 (*n* = 49) produced reliable signals in the applied real-time PCRs in relation to all DNA extraction protocols. Six of the 100 organ samples extracted with protocol 2 showed inhibition in the real-time PCR and generated no signals neither for ß-actin nor for Heli or IS 1081 and were therefore excluded from statistical analysis. From all other samples signals were detected even if the samples were previously stored at −20 °C.

### DNA extraction according to protocol 1 – MTC results

Results of protocol 1, which had been generated at the local Food and Health Safety Authority, were used as the reference method and as basic values for the comparison to the protocols 2 and 3. Efficiencies of the established DNA extraction protocols were determined by comparing their values to those generated with Protocol 1. Therefore, the decrease of Ct-values was regarded as an improvement for sensitivity for the different extraction methods. In protocol 1, 81.1 % of all bovine tissue samples (*n* = 74) were tested MTC negative (*n* = 60), 1.4 % had a questionable result (*n* = 1) and 17.5 % tested positive for MTC DNA (*n* = 13). In the case of red deer samples (*n* = 26) protocol 1 produced the following results: 57.7 % of the samples (*n* = 15) tested negative for MTC-specific DNA, while 38.5 % (*n* = 10) were positive and only 3.8 % (*n* = 1) were considered equivocal.

### DNA extraction according to protocol 2 – MTC results

Seventy-four bovine tissue samples were investigated by magnetic capture protocol and revealed reliable results by real-time PCR (see Table 2). The detection rate of MTC positive samples increased up to 24.3 % (*n* = 18) and consequently the detection rate for negative results decreased to 68.9 % (*n* = 51). The number of questionable results increased to 4.05 % (*n* = 3, animal no. 11, 13 and 20), which were positive in protocol 1. This was not unexpected since only suboptimal tissue samples were available for protocol 2 (leftovers sometimes without granuloma after official screening of the samples had been completed). Two samples (animal no. 18 and 23) were not evaluable with the new method (2.70 %) and they did not show any Ct-values, neither for ß-actin nor for Heli or IS 1081. However, these samples showed sample weights less than three grams.

Additionally, 26 tissue samples from red deer were screened by magnetic capture protocol and revealed evaluable results by real-time PCR (Table 3). In total 42.3 % of the samples (*n* = 11) tested positive for bTB. Furthermore, we found 15.4 % of the samples not evaluable as they did not produce any Ct-values neither for ß-actin nor for Heli or IS 1081 (*n* = 4; animal no. 8, 9, 10 and 16). These non-evaluable organs also had a weight less than three grams like mentioned above for cattle tissue samples which were unevaluable.

While the same number of red deer samples tested positive with both protocols (*n* = 10; 38.5 %), protocol 2 could detect one more sample as MTC positive, which was considered negative in protocol 1 (animal no. 11). Instead, one sample positive in protocol 1 was not evaluable with protocol 2 (animal no. 8). Animals no. 10 and 16 were not analyzable after magnetic capture and negative in protocol 1. Animal no.9 was tested negative after magnetic capture but considered equivocal with protocol 1.

### DNA extraction according to protocol 3 – MTC results

Thirty-eight of 74 bovine tissue samples were screened with an additional DNA extraction method, protocol 3. Protocol 3 increased the fraction of positive MTC results up to 31.6 % (*n* =12) and equivocal results increased from 2.6 % (*n* = 1, Table 2, animal no. 11) in protocol 1 to 7.9 % (*n* = 3, animal no. 11, 16, and 23) in protocol 3. In turn negative results decreased to 55.3 % (*n* = 21). However, Ct-values of doubtful samples appeared slightly above the detection limit at Ct = 39.00.

Furthermore, 11 of 26 tissue samples form red deer were tested according to protocol 3. Overall, 63.6 % of the sediments (*n* = 7) were negative for MTC DNA, while 36.4 % (*n* = 4, Table 3, animals no.11, 12, and 17) tested positive. With protocol 1 we found 72.7 % (*n* = 8) negative and only 27.3 % (*n* = 3) positive for MTC.

### Ct-values of the three different extraction methods for ß-actin

Protocol 1 and 3 resulted in average Ct_ß-actin_ = 21.61 and Ct_ß-actin_ = 21.56, respectively, whereas with protocol 2 we found less ß-actin with an average Ct_ß-actin_ = 27.24 (Additional file 2: Table S2). Average Ct-values for ß-actin in red deer tissue samples resembled those generated for cattle tissue samples: protocol 1 Ct_ß-actin_ = 20.85; protocol 2 Ct_ß-actin_ = 29.04; protocol 3 Ct_ß-actin_ = 20.30 (Additional file 3: Table S3).

### DNA quality and quantity

For protocol 2 an average value of 15.6 μg/ml was calculated for the DNA amount and DNA purity was on average at 1.70 (A260/A280). For protocol 3 DNA amount was on average at 281.4 μg/ml and DNA purity at 1.84 (A260/A280).

### Overall results per animal - comparison of protocol 1 and 2 (*n* = 34 cattle and *n* = 18 red deer)

In ten of 34 cattle, from which tissue samples were processed according to protocol 1, MTC-specific DNA was detected by real-time PCR in at least one of the 11 tissue samples. These animals were considered positive for bTB. No MTC-specific DNA was detected in the samples of the other 24 cattle examined in this study and consequently they were considered negative for bTB. According to the same criteria, six of 18 red deer were tested positive and 12 were tested negative for bTB. Usually, from red deer only one tissue sample per animal was available and was initially examined with protocol 1. The remains of this sample, which were in general very small, were then used for further processing with protocol 2 and 3. Protocol 2 revealed six more cattle as bTB-positive (animal no. 14, 21, 23, 25, 29 and 30) compared to protocol 1. Cattle no. 13 was classified as positive with protocol 1 and equivocal with protocol 2. However, the Ct-value for gene IS 1081 (Ct_IS 1081_ = 39.25) was only slightly above the detection threshold of Ct_IS 1081_ = 39.00. No cattle previously classified as bTB-positive with protocol 1 was classified negative with protocol 2. In the case of red deer samples, protocol 2 allowed five animals to be classified as bTB-positive and 11 animals as bTB-negative. Two red deer could not be categorized with protocol 2, because only one small tissue sample per animal was available for further testing and both did not yield evaluable results. Therefore, a DNA extraction with protocol 2 was performed.

### Comparison of protocol 1, 2 and 3 (*n* = 23 cattle and *n* = 9 red deer)

Selected samples from nine of all 18 red deer cases and 23 of all 34 bovine cases were further processed according to protocol 3. Five cattle and three red deer cases had been classified as bTB-positive, while 18 cattle and six red deer were classified as negative by using protocol 1. In contrast the employment of protocol 2 and 3 yielded in ten bTB-positive bovine cases, while protocol 3 produced an additional equivocal result (Table 2, animal no. 23). In this case, Ct_IS1081_ was 40.23, so only slightly above the detection limit. Twelve cattle were classified negative by these two methods. Red deer testing with protocol 3 produced the same results as protocol 1: three cervids were bTB-positive and six negative. As with protocol 2 two samples from red deer cases could not be analyzed and no sample material was left to repeat the analysis.

### Statistical analysis - kappa index

The three extraction protocols resulted in a moderate (0.52; protocol 1 vs 3) to almost perfect agreement (1.00; red deer sample testing with all protocols). The calculated values are shown in Table 4.

**Table 4:** Diagnostic efficiency of the three DNA extraction protocols: agreement between tests (Kappa index, k)

Species	Reference	Agreement (k)	Interpretation
Cattle Table 2	P1 vs P2	0.621	Substantial agreement
	P1 vs P3	0.522	Moderate agreement
	P2 vs P3	0.789	Substantial agreement
Red deer Table 3	P1 vs P2	1.00	almost perfect agreement
	P1 vs P3	1.00	almost perfect agreement
	P2 vs P3	1.00	almost perfect agreement

#### Mycobacterial cultivation

A total number of 16 animals were tested by culture in this study. Culture material was obtained from ten cattle and six red deer cases, which had tested bTB-positive by real-time PCR. Samples showed bacterial growth after four to eight weeks of incubation on solid and/or liquid media. Single colonies from these cultures were used for species identification and were all confirmed as *M. caprae* by GenoType MTBC (Hain Lifescience, Nehren, Germany; data not shown).

#### Evaluation of the Ziehl-Neelsen staining of the sediments

**Fig. 1:**
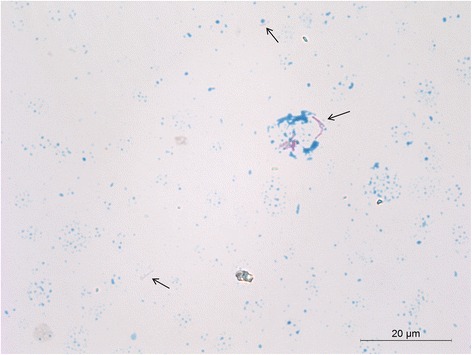
Picture of a Ziehl-Neelsen stained sediment. The arrows indicate the presence of acid fast bacilli

Acid fast bacilli were clearly visible (Fig. 1) when two Ziehl-Neelsen stained sediments that showed low Ct-values for Heli and IS 1081 in protocol 3 were evaluated microscopically (animal no. 18, Table 2 and no. 17, Table 3). Viability of the microbes was not assessed. Microscopic examination of two sediments considered MTC-negative by PCR provided no evidence of acid fast bacteria (animal no. 7 and 22, Table 2).

## Discussion

The chronic course of mycobacterial infection leads to long incubation periods, where infected individuals remain clinically asymptomatic. In animals shedding of mycobacteria before clinical manifestation of tuberculosis is critical and thus controlled surveillance is an essential instrument to control the spread of the mycobacteria [25, 26]. Due to the lack of clinical signs, diagnostic procedures for the detection of bTB in cattle are challenging, expensive and currently are based on an authority regulated prescribed protocol. Cellular immune reactions as represented by the tuberculin skin test, preferably the SICCT or the interferon gamma release test, indicate the infectious status of an animal. The in vivo immune response should be confirmed post mortem by direct detection of the pathogen or its DNA using PCR and/or culture isolation [18, 19]. For DNA extraction of a template for subsequent PCR, only small amounts of tissue (preparations up to a maximum of 1 g, hence 200–400 μl for DNA extraction) are commonly used. This may lead to false negative results due to the well-known inhomogeneous distribution of mycobacteria in tissue material [27]. In addition, animals infected in an early pre-granuloma forming status can contain macroscopically normal but infected organs (NVL, non-visible lesions). This situation makes it impossible to recognize mycobacterial presence and location in the organ to allow targeted MTC-specific DNA extraction. Consequently, DNA extraction methods, which improve the sensitivity for mycobacterial DNA detection, are urgently needed [17, 27]. It was hypothesized that this problem might be solved when large sample volumes are processed and MTC-specific DNA can be concentrated and captured in the sample material. A magnetic capture protocol established in this study (protocol 2) was proven to be a sufficient method for MTC DNA detection by the use of artificially spiked lymph nodes.

This protocol revealed stable results with the MTC real-time PCR. However, it was not possible to define the detection limit of this method due to the fact that pathogenic mycobacteria have a hydrophobic and waxy mycolic acid layer in the cell wall so that the bacteria can attach to each other [28]. Consequently, separation and accurate quantification of these pathogens by bacterial count is not possible [29]. A reliable quantification on the gene copy number of Heli or IS 1081 was also not possible, because *M. caprae* seems to exhibit substantial genetic variations within its genome as described for the RD4 region, and also possibly virulence related regions [30, 31]. Furthermore, as little information is available in the literature concerning the exact number of gene copies for IS 1081 [32] and Heli [33–35] it was not possible to define the detection limit on the basis of gene copy numbers in the analyzed *M. caprae* subtypes. Instead, the decrease in Ct-values was chosen as the parameter that defines an improvement of the extraction protocol.

By extracting DNA from field samples of naturally infected cattle and red deer with protocol 2 and subsequently generating reliable and evaluable results with the real-time PCR for ß-actin, Heli and IS 1081 we did proof that with the new protocol we cannot only efficiently extract DNA from tissue samples containing high amounts of bacteria such as artificially spiked lymph nodes for establishing the new protocol, but also from samples containing low numbers of bacteria such as field samples from naturally infected animals. Organs with NVL are the most common problems when only small sample volumes are used for culture and DNA extraction [12, 36]. We were able to show that artificially spiked lymph nodes (*M. bovis* BCG) processed with protocol 2 resulted in a 100 % positive MTC detection rate. However, only 40 % of the artificially spiked lymph nodes revealed positive results when samples were processed using the prescribed DNA extraction protocol (protocol 1). These results underline that the use of small sample sizes (~20 to 40 mg) decreases the probability for targeted DNA extraction and can lead to false negative results. Using the DNA extraction protocol 1 a reasonable number of bovine and red deer field samples (*n* = 100) were tested as equivocal or even negative that later on had been confirmed to contain *M. caprae* by culture isolation. On the other hand, using a larger sample mass, more samples were found to be positive with the magnetic capture protocol (*n* = 23 with protocol 1; *n* = 28 with protocol 2). These results confirmed this method to be more sensitive compared to protocol 1. Since the established magnetic capture protocol specifically enriches MTC DNA, the procedure is useful especially when only small numbers of target genes are expected in organs like NVL organs. Samples, that had to be qualified as equivocal or negative using protocol 1 turned out to be positive (*n* = 5) or to a lesser extend still equivocal (*n* = 2) when protocol 2 was applied. We even assume that more samples would have been assessed as *M. caprae* positive, when one considers that suboptimal tissue samples – only remnant tissues after protocol 1 had been available – had to be processed for application of protocol 2. Leftover material most likely did not contain mycobacteria anymore, because granulomas were largely used for the officially prescribed diagnostic methods. Furthermore, in most cases not all 11 tissue samples of the bTB suspicious animal were available and again we were only able to investigate the remnant organ tissue sets. This explains why samples that had been tested positive for bTB (protocol 1) generated only equivocal or even negative results when protocol 2 was applied (Table 2, No. 67). In six out of 100 organ samples we were not able to generate Ct- values, neither for ß-actin nor for Heli or IS 1081. These samples weighed less than 3 g. In these cases we speculate that the applied proteinase K concentration was probably too high and the released DNA was digested or otherwise destroyed. Consequently, protocol 2 should only be used for a sample mass larger than 4 g or proteinase concentration should be adjusted to the small sample volumes. Nevertheless, for all samples above 4 g the results presented in this study show that the magnetic capture protocol is a valuable tool to improve accessibility to mycobacteria inhomogeneously distributed in sample material. In addition, MTC-specific capture oligonucleotides ensure the target-oriented fishing of MTC DNA onto magnetic particles and allow inhibitory substances and non-target DNA to be washed away and removed. This assumption was proved by the decrease of ß-actin Ct- values from of around Ct_ß-actin_ = 21.00 in protocols 1 and 3 to Ct_ß-actin_ = 27.00 in protocol 2 and also by the yarding DNA amounts generated with the different protocols. The high amounts of overall DNA obtained with protocol 3 - and probably also with the similar protocol 1 - are not surprising, because MTC-specific DNA was not concentrated and large amounts of host DNA is present in the samples. Interestingly, the purity of DNA generated with protocol 3 is slightly better compared to the DNA obtained with the magnetic capture protocol, probably a result of the efficient purification process on a silica-based matrix. These findings convincingly demonstrate that the specific capture oligonucleotides efficiently eliminate host DNA while MTC-specific DNA remains in the solution for further processing. Consequently, inhibitory substances such as collagen and hematin [37] can be reduced and unevaluable PCR-results, which may be obtained due to inhibitory substances, can be avoided. The magnetic capture method, however, is labor-intensive and time consuming compared to protocol 1 and should be considered as not suitable for daily use in routine diagnostics. To overcome these problems, pellets of the homogenized and digested material (*n* = 49) were further processed with protocol 1, which led to a time reduction of about 3 h compared to protocol 2. The time needed for DNA extraction with protocol 3 is similar to the time needed for DNA extraction with the current used protocol (protocol 1). The modified method allows the simultaneous processing of several samples. In contrast, to the magnetic capture protocol there is no limitation concerning the sample size (samples < 3 g), a fact that may have led to the highest proportion of positive PCR results (protocol 3 and 2, *n* = 16; protocol 1, *n* = 11) in comparison to the other two protocols. However, when small bacterial loads or inhibitory substances due to lytic tissue material are expected, the more time consuming magnetic capture protocol (protocol 2) is recommended as no target DNA is concentrated with protocol 3. DNA release from mycobacterial cells and DNA concentration is critical as successful molecular detection largely depends on the efficiency of the DNA extraction method [38]. Cell wall disruption can be achieved in various ways like mechanical disruption, enzymatic or chemical lysis or a combination of these methods. We chose a combination of mechanical disruption by bead-beating (FastPrep 96 machine) in combination with and an enzymatic digestion (proteinase K) as satisfactory results were achieved in previous studies [39, 40]. Additionally, digestion time was increased to an overnight-incubation step and subjoined by two inactivation steps for 15 min at 99 °C. However, destruction of all acid-fast bacilli was not efficient when these modifications were applied as several nests of bacteria were still visible after Ziehl-Neelsen staining of the sediments. Mycobacteria were detectable in nests or as single red rods, some continuously and others discontinuously dyed. We expect that the bacilli were not infectious anymore and that the cell wall was damaged in such a way that MTC-specific DNA was released from the bacilli and similar results were obtained with protocol 3 as with protocol 2.

We compared the three different extraction methods at two different levels. At the first level, we compared the Ct-values per tissue sample so that we were able to make accurate statements whether MTC-specific Ct-values increased or a decreased when a specific extraction method was applied. From the epidemiological point of view, the most important unit to consider is the animal. So we compared at the second level the three extraction methods on the basis of the overall result per animal. When a single sample of the 11 tissue samples collected per animal is positive for MTC-specific DNA, the animal is considered bTB-positive. The kappa indices for comparison of the three DNA extraction protocols were calculated at this level. Culture as the gold standard for bTB diagnostics, could not be included for the purpose of comparison because tissue samples had been selected based on their positive or equivocal PCR status and were then further processed for cultivation. Hence, protocol 1 was chosen as reference method to compare the results produced with the three extraction methods.

Since the tissue samples used for this project were incomplete due to preceding testing, we cannot make any statement about the infection frequency in relation to target organs. Most frequently intestinal and lung lymph nodes together with parts of lungs responded positive for *M. caprae* in this study. If kidney or liver were positive for MTC-specific DNA, it was most likely that intestine and lungs were also affected. To limit work time and costs, it would be preferable to select only the most susceptible organs or to pool suspicious organs for further processing in accordance with protocol 2. As PCR testing of tissue samples with NVL in combination with the inhomogeneous distribution of *Mycobacteria* spp. and the small sample size that is used in the prescribed protocol may result in false negative results, the newly developed DNA extraction methods allow the investigation of a larger sample volume for DNA extraction and therefore increase the probability to detect MTC-specific DNA in animal tissue.

## Conclusion

Magnetic capture is a robust and highly sensitive method for targeted MTC- specific DNA detection in larger tissue masses, especially when a small inhomogeneous bacterial load is expected or if only autolytic sample material is available. For daily routine diagnostics, protocol 3 (sediment screening of large sample volumes) is recommended as processing of large sample masses can be performed in a short time. Both newly established protocols offer reliable MTC-specific DNA extraction from larger tissue masses, which increases the detection rate in subsequent PCR protocols compared to the commonly used small tissue pieces.

## Additional files

Additional file 1: Table S1. Reagents used for tissue processing in relation to the tissue mass. (DOC 43 kb) Additional file 2: Table S2. Ct-values of ß-actin DNA detection in field tissue samples of cattle achieved in the three DNA extraction protocols. (DOC 157 kb) Additional file 3: Table S3. Ct-values of ß-actin DNA detection in field tissue samples of red deer achieved in the three DNA extraction protocols. (DOC 74 kb)
