# Supplementary material for: Two alternative DNA extraction methods to improve the detection of Mycobacterium-tuberculosis-complex members in cattle and red deer tissue samples
Source: BMC Microbiol. 2016 Sep 15;16:213. doi: 10.1186/s12866-016-0816-2 (PMC5024493; doi:10.1186/s12866-016-0816-2)
Supplement: Additional file 1: Table S1. — Reagents used for tissue processing in relation to the tissue mass. (DOC 43 kb) [file 12866_2016_816_MOESM1_ESM.doc]

**Additional file 1: Table S**1 Reagents used for tissue processing in relation to the tissue mass

| tissue sample weight in g | proteinase K in µl | ATL buffer in ml |
| --- | --- | --- |
| 1 | 15 | 2.3 |
| 2 | 30 | 4.7 |
| 3 | 45 | 7.0 |
| 4 | 60 | 9.3 |
| 5 | 75 | 11.7 |
| 6 | 90 | 14.0 |
| 7 | 105 | 16.3 |
| 8 | 120 | 18.6 |
| 9 | 135 | 21.0 |
| 10 | 150 | 23.3 |
| 11 | 165 | 25.6 |
| 12 | 180 | 28.0 |
| 13 | 195 | 30.3 |
| 14 | 210 | 32.6 |
| 15 | 225 | 33.0 |

| volume of tissue digest per 15-ml tube in ml | volume of Streptavidin sepahrose per 15-ml tube in µl | volume of 5 M NaCl solution per 15-ml tube in ml |
| --- | --- | --- |
| 14 | 55 |  |
| 13 | 55 |  |
| 12 | 55 |  |
| 11 | 55 |  |
| 10 | 55 | 2.0 |
| 9 | 50 | 1.8 |
| 8 | 45 | 1.6 |
| 7 | 40 | 1.4 |
| 6 | 35 | 1.2 |
| 5 | 30 | 1.0 |
| 4 | 25 | 0.8 |
| 3 | 20 | 0.6 |
| 2 | 15 | 0.4 |
| 1 | 10 | 0.2 |
