# Supplementary material for: Two alternative DNA extraction methods to improve the detection of Mycobacterium-tuberculosis-complex members in cattle and red deer tissue samples
Source: BMC Microbiol. 2016 Sep 15;16:213. doi: 10.1186/s12866-016-0816-2 (PMC5024493; doi:10.1186/s12866-016-0816-2)
Supplement: Additional file 2: Table S2. — Ct-values of ß-actin DNA detection in field tissue samples of cattle achieved in the three DNA extraction protocols. (DOC 157 kb) [file 12866_2016_816_MOESM2_ESM.doc]

**Additional file 2: Table S2 Ct-values of ß-actin DNA detection in field tissue samples of cattle achieved in the three DNA extraction protocols**

| Animal No. | Tissue | Protocol 1 | | | Protocol 2 | | | Protocol 3 | | |
| --- | --- | --- | --- | --- | --- | --- | --- | --- | --- | --- |
|  |  | ß-Actin Heli | ß-Actin IS 1081 | ß-Actin average | ß-Actin Heli | ß-Actin IS 1081 | ß-Actin average | ß-Actin Heli | ß-Actin IS 1081 | ß-Actin average |
| 1 | Lung | 20.06 | 19.96 | 20.01 | 27.90 | 27.56 | 27.73 | - | - | - |
|  | Diaphragm | 21.31 | 21.13 | 21.22 | 26.44 | 26.50 | 26.47 | - | - | - |
| 2 | Lung | 23.09 | 22.61 | 22.85 | 26.94 | 26.90 | 26.92 | - | - | - |
| 3 | Intestinal ln | 19.08 | 18.97 | 19.03 | 24.24 | 24.38 | 24.31 | - | - | - |
|  | Kidney | 20.63 | 20.79 | 20.71 | 25.90 | 25.58 | 25.74 | 19.06 | 19.13 | 19.10 |
|  | Kidney ln | 18.53 | 18.47 | 18.50 | 23.38 | 23.59 | 23.49 | 18.93 | 18.85 | 18.89 |
| 4 | Intestinal ln | 18.78 | 18.69 | 18.74 | 29.43 | 29.53 | 29.48 | - | - | - |
|  | Liver | 20.36 | 19.87 | 20.12 | 28.30 | 28.27 | 28.29 | 21.42 | 21.22 | 21.32 |
| 5 | Ln cervicalis superf. | 19.61 | 19.00 | 19.31 | 29.94 | 29.78 | 29.86 | 20.93 | 20.97 | 20.95 |
|  | Mediastinal ln | 21.88 | 21.74 | 21.81 | 25.85 | 25.96 | 25.91 | 20.62 | 20.59 | 20.61 |
|  | Kidney | 24.31 | 24.02 | 24.17 | 28.76 | 28.66 | 28.71 | - | - | - |
|  | Kidney ln | 21.86 | 22.06 | 21.96 | 30.59 | 30.60 | 30.59 | - | - | - |
| 6 | Liver | 21.85 | 21.73 | 21.79 | 31.23 | 30.89 | 31.06 | - | - | - |
|  | Lung | 23.93 | 24.03 | 23.98 | 21.38 | 21.25 | 21.32 | 22.28 | 22.26 | 22.27 |
|  | Kidney | 24.31 | 23.71 | 24.01 | 25.75 | 25.59 | 25.67 | - | - | - |
| 7 | Lung | 22.72 | 23.14 | 22.93 | 26.40 | 26.52 | 26.46 | - | - | - |
| 8 | Lung ln | 19.61 | 20.36 | 19.99 | 29.09 | 29.15 | 29.12 | - | - | - |
| 9 | Retropharyngeal ln | 19.24 | 19.99 | 19.62 | 23.29 | 23.00 | 23.15 | 20.15 | 20.75 | 20.45 |
| 10 | Intestinal ln | 17.60 | 18.03 | 17.82 | 24.37 | 24.24 | 24.30 | - | - | - |
| 11 | Liver ln | 18.68 | 17.70 | 18.19 | 25.61 | 25.39 | 25.50 | 21.08 | 20.98 | 21.03 |
|  | Lung | 18.81 | 17.77 | 18.29 | 22.05 | 22.34 | 22.20 | 20.64 | 20.42 | 20.53 |
| 12 | Intestinal ln | 18.78 | 19.93 | 19.36 | 25.68 | 25.65 | 25.67 | - | - | - |
| 13 | Intestinal ln | 17.90 | 18.64 | 18.27 | 29.58 | 29.83 | 29.70 | - | - | - |
| 14 | Intestinal ln | 24.40 | 24.05 | 24.23 | 27.58 | 27.28 | 27.43 | 20.13 | 20.04 | 20.09 |
| 15 | Diaphragm | 22.81 | 22.81 | 22.81 | 30.34 | 30.09 | 30.22 | 23.36 | 22.97 | 23.17 |
|  | Diaphragm | 24.10 | 24.10 | 24.10 | 29.93 | 29.91 | 29.92 | 24.33 | 24.28 | 24.31 |
|  | Diaphragm | 18.78 | 19.64 | 19.21 | 26.01 | 26.18 | 26.09 | - | - | - |
| 16 | Intestinal ln | 22.45 | 22.37 | 22.41 | 32.15 | 31.20 | 31.68 | 20.98 | 21.15 | 21.65 |
|  | Lung | 23.48 | 23.63 | 23.56 | 44.01 | 41.55 | 42.78 | 23.23 | 23.36 | 23.30 |
|  | Lung ln | 19.65 | 19.57 | 19.61 | 22.22 | 22.28 | 22.25 | 21.43 | 21.52 | 21.48 |
|  | Spleen | 21.80 | 21.75 | 21.78 | 20.23 | 20.07 | 20.15 | 21.09 | 20.95 | 21.02 |
|  | Kidney | 21.14 | 20.65 | 20.90 | 23.02 | 23.09 | 23.06 | 20.03 | 20.41 | 20.22 |
| 17 | Intestinal ln | 19.86 | 19.81 | 19.84 | 25.40 | 25.36 | 25.38 | - | - | - |
| 18 | Liver | 23.47 | 23.10 | 23.29 | 32.03 | 32.29 | 32.16 | - | - | - |
|  | Lunge | 21.33 | 20.76 | 21.05 | 20.80 | 20.53 | 20.66 | 20.72 | 20.84 | 20.78 |
|  | Mediastinal ln | 19.35 | 20.73 | 20.04 | 26.46 | 25.53 | 26.00 | 21.28 | 21.57 | 21.42 |
|  | Spleen | 22.42 | 22.15 | 22.29 | 24.28 | 24.55 | 24.42 | - | - | - |
|  | Kidney | 24.17 | 24.83 | 24.50 | 25.77 | 25.74 | 25.76 | - | - | - |
|  | Retropharyngeal ln | 20.62 | 20.40 | 20.51 | no ct | no ct | no ct | - | - | - |
| 19 | Mediastinal ln | 21.52 | 20.57 | 21.05 | 28.29 | 28.25 | 28.27 | 20.28 | 20.17 | 20.23 |
| 20 | Intestinal ln | 20.63 | 19.88 | 20.26 | 31.04 | 31.05 | 31.05 | - | - | - |
|  | Mediastinal ln | 18.15 | 19.02 | 18.59 | 30.67 | 31.14 | 30.90 | - | - | - |
|  | Retropharyngeal ln | 18.64 | 20.08 | 19.36 | 25.72 | 25.62 | 25.67 | - | - | - |
| 21 | Lung ln | 21.51 | 21.41 | 21.46 | 26.11 | 26.20 | 26.16 | 20.16 | 19.93 | 20.05 |
| 22 | Intestinal ln | 23.41 | 23.91 | 23.66 | 26.16 | 26.27 | 26.21 | 22.78 | 22.36 | 22.57 |
|  | Liver | 20.54 | 20.02 | 20.28 | 32.91 | 33.09 | 33.00 | 21.79 | 21.27 | 21.53 |
|  | Lung | 24.40 | 24.39 | 24.40 | 27.71 | 27.63 | 27.67 | - | - | - |
|  | Lung ln | 22.25 | 22.03 | 22.14 | 32.02 | 32.24 | 32.13 | - | - | - |
|  | Spleen | 20.16 | 19.80 | 19.98 | 24.94 | 25.18 | 25.06 | - | - | - |
|  | Kidney | 22.11 | 22.81 | 22.46 | 29.20 | 29.47 | 29.34 | - | - | - |
|  | Retropharyngeal ln | 19.93 | 19.90 | 19.92 | 23.15 | 23.23 | 23.19 | 23.78 | 23.61 | 23.70 |
| 23 | Kidney | 24.72 | 23.85 | 24.29 | no ct | no ct | no ct | 22.88 | 22.83 | 22.86 |
|  | Muscle | 27.98 | 26.81 | 27.40 | 29.47 | 29.29 | 29.38 | 23.82 | 23.87 | 23.85 |
|  | Palatine tonsil | 23.58 | 22.49 | 23.04 | 29.47 | 29.29 | 29.38 | 20.45 | 20.53 | 20.49 |
| 24 | Udder ln | 22.55 | 21.48 | 22.02 | 26.45 | 26.43 | 26.44 | 19.35 | 19.30 | 19.33 |
| 25 | Intestinal ln | 18.90 | 18.25 | 18.58 | 29.43 | 30.34 | 29.89 | 23.00 | 23.16 | 23.08 |
| 26 | Lung ln | 24.03 | 26.22 | 25.13 | 25.14 | 24.99 | 25.06 | - | - | - |
|  | Rumen | 25.26 | 28.83 | 27.05 | 27.38 | 27.25 | 27.31 | 22.90 | 22.88 | 22.89 |
| 27 | Lung | 24.70 | 22.64 | 23.67 | 32.81 | 32.68 | 32.74 | - | - | - |
|  | Thymus | 23.45 | 21.20 | 22.33 | 26.57 | 26.44 | 26.50 | 20.38 | 20.56 | 20.47 |
| 28 | Lung | 23.85 | 22.85 | 23.35 | 25.42 | 24.94 | 25.18 | - | - | - |
| 29 | Pelvic ln | 24.02 | 25.03 | 24.53 | 31.24 | 31.15 | 31.20 | - | - | - |
|  | Mesentery | 23.12 | 23.14 | 23.13 | 22.88 | 22.47 | 22.67 | 23.44 | 23.65 | 23.54 |
|  | Kidney | 21.59 | 21.87 | 21.73 | 27.02 | 26.87 | 26.95 | - | - | - |
|  | Perintoneum | 23.46 | 21.35 | 22.41 | 26.13 | 26.05 | 26.09 | 23.96 | 28.09 | 26.02 |
| 30 | Intestinal ln | 18.69 | 16.42 | 17.56 | 25.66 | 25.53 | 25.60 | 21.50 | 21.48 | 21.49 |
|  | Liver ln | 22.73 | 22.89 | 22.81 | 26.56 | 26.65 | 26.61 | - | - | - |
|  | Lung ln | 19.66 | 20.42 | 20.04 | 28.92 | 28.76 | 28.84 | - | - | - |
|  | Retropharyngeal ln | 17.58 | 19.90 | 18.74 | 23.18 | 23.16 | 23.17 | - | - | - |
| 31 | Kidney | 19.72 | 21.83 | 20.78 | 26.63 | 26.38 | 26.51 | - | - | - |
| 32 | Lung | 21.14 | 20.96 | 21.05 | 29.38 | 29.49 | 29.43 | 20.24 | 20.24 | 20.24 |
|  | Lung ln | 21.28 | 20.46 | 20.87 | 28.45 | 28.46 | 28.46 | - | - | - |
| 33 | Lung ln | 24.59 | 24.74 | 24.67 | 28.09 | 28.22 | 28.15 | - | - | - |
| 34 | Spleen | 28.89 | 26.99 | 27.94 | 27.71 | 27.73 | 27.72 | 21.84 | 21.80 | 21.82 |

- not done

ln lymph node
