# Supplementary material for: Two alternative DNA extraction methods to improve the detection of Mycobacterium-tuberculosis-complex members in cattle and red deer tissue samples
Source: BMC Microbiol. 2016 Sep 15;16:213. doi: 10.1186/s12866-016-0816-2 (PMC5024493; doi:10.1186/s12866-016-0816-2)
Supplement: Additional file 3: Table S3. — Ct-values of ß-actin DNA detection in field tissue samples of red deer achieved in the three DNA extraction protocols. (DOC 74 kb) [file 12866_2016_816_MOESM3_ESM.doc]

**Additional file 3: Table S3 Ct-values of ß-actin DNA detection in field tissue samples of red deer achieved in the three DNA extraction protocols**

| Animal No. | Tissue | Protocol 1 | | | Protocol 2 | | | Protocol 3 | | |
| --- | --- | --- | --- | --- | --- | --- | --- | --- | --- | --- |
|  |  | ß-Actin Heli | ß-Actin IS 1081 | ß-Actin average | ß-Actin Heli | ß-Actin IS 1081 | ß-Actin average | ß-Actin Heli | ß-Actin IS 1081 | ß-Actin average |

| 1 | Intestinal ln | 18.92 | 19.13 | 19.03 | 29.98 | 29.96 | 29.97 | - | - | - |
| --- | --- | --- | --- | --- | --- | --- | --- | --- | --- | --- |
| 2 | Intestinal ln | 19.19 | 19.01 | 19.10 | 44.02 | 44.51 | 44.26 | 20.62 | 20.71 | 20.67 |
| 3 | Mesenteric ln | 20.83 | 20.84 | 20.84 | 25.77 | 25.85 | 25.81 | - | - | - |
| 4 | Intestinal ln | 21.18 | 20.80 | 20.99 | 27.02 | 26.52 | 26.77 | - | - | - |
| 5 | Intestinal ln | 23.36 | 23.02 | 23.19 | 27.39 | 27.20 | 27.30 | - | - | - |
| 6 | Peritoneum | 19.89 | 20.43 | 20.16 | 26.47 | 26.44 | 26.45 | - | - | - |
| 7 | Intestinal ln | 18.51 | 17.77 | 18.14 | 31.13 | 31.20 | 31.16 | 19.92 | 19.75 | 19.84 |
| 8 | Mesenteric ln | 19.53 | 19.52 | 19.53 | no ct | no ct | no ct | - | - | - |
| 9 | Intestinal ln | 19.30 | 19.02 | 19.16 | no ct | no ct | no ct | - | - | - |
|  | Lung | 18.33 | 18.34 | 18.34 | 26.44 | 26.25 | 26.35 | - | - | - |
| 10 | Intestinal ln | 22.12 | 22.64 | 22.38 | 25.62 | 25.58 | 25.60 | - | - | - |
|  | Lung | 26.25 | 26.83 | 26.54 | no ct | no ct | no ct | - | - | - |
| 11 | Intestinal ln | 21.44 | 20.40 | 20.92 | 30.47 | 30.37 | 30.42 | 20.37 | 20.27 | 20.32 |
|  | Retropharyngeal ln | 19.72 | 19.48 | 19.60 | 23.28 | 23.38 | 23.33 | 19.35 | 19.30 | 19.32 |
| 12 | Intestinal ln | 22.21 | 21.29 | 21.75 | 27.62 | 27.93 | 27.77 | 22.35 | 22.57 | 22.46 |
|  | Retropharyngeal ln | 19.16 | 19.18 | 19.17 | 31.99 | 32.00 | 32.00 | 20.04 | 19.70 | 19.87 |
| 13 | Lung | 20.72 | 19.38 | 20.05 | 28.65 | 28.58 | 28.62 | - | - | - |
|  | Pleura | 21.78 | 21.30 | 21.54 | 26.10 | 26.31 | 26.20 | - | - | - |
|  | Palatine tonsil | 19.35 | 19.86 | 19.61 | 25.38 | 25.32 | 25.35 | - | - | - |
|  | Mass | 23.57 | 23.26 | 23.42 | 26.77 | 26.71 | 26.74 | - | - | - |
|  | Diaphragm | 18.86 | 18.16 | 18.51 | 32.63 | 32.69 | 32.66 | - | - | - |
| 14 | Lung | 21.19 | 20.61 | 20.90 | 21.94 | 22.06 | 22.00 | 20.36 | 20.74 | 20.55 |
| 15 | Retropharyngeal ln | 21.37 | 21.52 | 21.45 | 33.93 | 33.78 | 33.86 | 19.64 | 19.91 | 19.78 |
| 16 | Intestinal ln | 23.93 | 24.04 | 23.99 | no ct | no ct | no ct | 21.73 | 21.37 | 21.55 |
| 17 | Retropharyngeal ln | 18.54 | 26.79 | 22.67 | 28.43 | 28.40 | 28.42 | 18.54 | 18.67 | 18.61 |
| 18 | Intestinal ln | 21.55 | 20.57 | 21.06 | 42.80 | 32.79 | 37.79 | 20.13 | 20.48 | 20.31 |

- not done

ln lymph node
